# Application of a Nociceptive Test Battery to Assess Potential Synergy between Two Analgesics in Healthy Subjects

**DOI:** 10.1021/acsptsci.4c00696

**Published:** 2025-02-14

**Authors:** Wouter Alexander Bakker, Monir Bertayli, Daniël Benjamin Dumas, Jeroen Elassaiss-Schaap, Maria Joanna Juachon, Karen Broekhuizen, Hemme Jacob Hijma, Geert Jan Groeneveld

**Affiliations:** † Centre for Human Drug Research, Leiden 2333 CL, The Netherlands; ‡ Leiden University Medical Centre, Leiden 2333 ZA, The Netherlands; § PD-Value, Utrecht 3584 CL, The Netherlands

**Keywords:** opioids sparing, chronic pain, translational, cognitive tests, evoked pain tests, pEEG

## Abstract

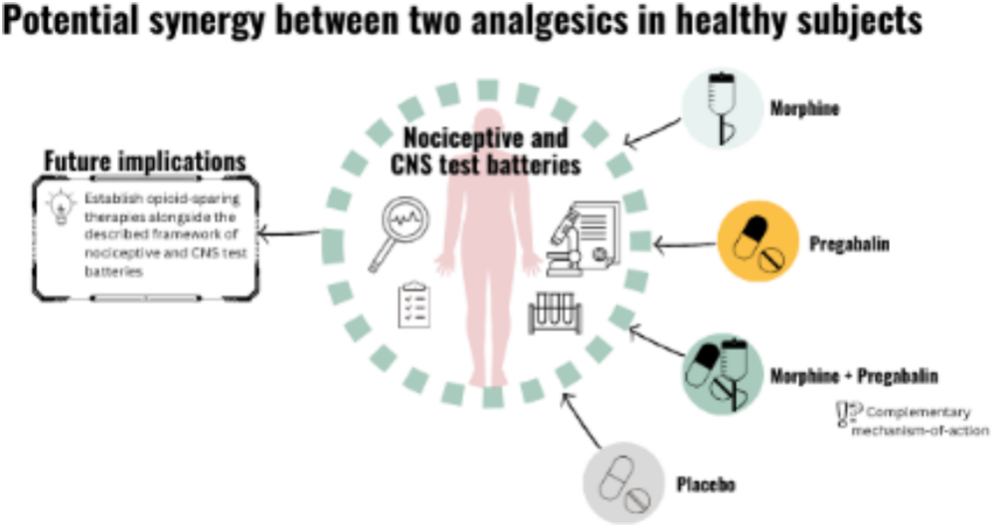

Chronic pain management remains a major challenge due to the risks associated with conventional treatments, such as opioids and NSAIDs, which carry significant risks, including addiction, tolerance, and adverse side effects, particularly with prolonged use. Combining opioid with nonopioid drugs offer a potential solution, as it may minimize opioid-related side effects by reducing the required opioid dose. We performed a study to compare the analgesic effects and safety of a pregabalin–morphine combination to each drug alone and placebo in healthy volunteers. A randomized, double-blind, placebo-controlled crossover design was used, with subjects receiving 300 mg of pregabalin combined with 3 and 7 mg of morphine, morphine only, pregabalin only, or a double placebo. Analgesic effects and CNS side effects were assessed up to 10 h postdose using nociceptive and neurocognitive test batteries. Results demonstrated that the pregabalin–morphine combination significantly increased pain tolerance compared to either drug alone on several pain tests (cold pressor, electrical burst, electrical stair, and pressure pain) with only minimal additional CNS side effects compared to monotherapy and placebo. This study indicates that validated nociceptive and CNS test batteries were suitable to assess the potential of opioid–sparing combination therapies in an experimental setting.

Chronic pain is one of the most prevalent medical conditions in the Western world. Approximately, 20% of the European population experiences chronic pain, resulting in a considerable impact on the healthcare system.^1,2^ Chronic pain management poses a challenge on healthcare professionals and researchers, as common treatments like opioids and NSAIDs carry significant risks, including addiction, tolerance, and adverse side effects, especially with long-term use.^3−6^ Therefore, safer, alternative approaches are needed for effective pain relief.^7^ Most neuropathic pain drugs are approved as monotherapies, but they provide only modest pain relief and often have dose-limiting side effects.^8−11^ Opioids, for example, have proven efficacy in reducing nociceptive pain and mixed pain such as in cancer but may also induce unacceptable side effects.^12^ A substantial group of patients treated with oral morphine suffer from excessive adverse effects, inadequate analgesia, or a combination of both.^13^ Reducing the opioid dosage might help alleviating adverse effects while preserving pain relief, thus lessening the impact of pain on both individuals and society.^3^

Combining different analgesic compounds, particularly an opioid with a nonopioid, is one approach to achieve improved analgesic effects. Opioid–nonopioid drug combinations are of special interest as they are anticipated to enhance pain relief through complementary action mechanisms while minimizing opioid-associated side effects by reducing the required opioid dose.^14^ Adding a nonopioid analgesic to an opioid may produce additive or synergistic effects, depending on the combination, representing a new approach to pain management. This approach shows promise in enhancing pain relief at lower opioid doses, potentially reducing adverse effects and addiction risks while addressing the pressing opioid crisis, which remains a significant global health challenge, driven by the widespread misuse, addiction, and risks of dependence and overdose associated with opioid medications.^15^ Until now, the literature is discrepant on which combination may be of most benefit for chronic pain patients. Two systematic reviews have been conducted but did not provide conclusive evidence in favor or against the use of opioid–sparing drugs. Both reviews highlighted the need for more robust, placebo-controlled randomized trials.^16,17^

The Horizon2020 QSPainRelief consortium (H2020-SC1-BHC-2018-2020) consists of multiple research groups investigating opioid–nonopioid drug combinations for improved analgesic effects and reduced adverse effects. This consortium’s approach is a full translational program: from *in silico* modeling via *in vitro* models to healthy volunteer studies and eventually studies in patients with (chronic) pain. In the consortium, morphine and pregabalin were selected for an opioid–nonopioid drug combination. Morphine, a cornerstone in cancer pain treatment,^18−21^ was chosen as the opioid to test in this study as it has been extensively characterized in both *in vitro* and *in vivo* studies, making it ideal for modeling purposes. Pregabalin, recommended as a first-line treatment for neuropathic pain, is a promising candidate for combination therapy with morphine. Studies have demonstrated the efficacy of pregabalin monotherapy in neuropathic pain patients, and early investigations into the pregabalin–morphine combination suggest that it may effectively manage neuropathic pain.^12−17^ However, many of these studies lacked placebo-controlled designs, were underpowered, or focused on postoperative or cancer-related pain. Thus, further placebo-controlled RCTs are needed to confirm the efficacy of the pregabalin–morphine combination for chronic pain.

We performed a randomized, double-blind, placebo-controlled crossover study to evaluate the analgesic effects of a nonopioid (pregabalin) and an opioid analgesic (morphine), as a combination therapy and monotherapy, in healthy volunteers. Pharmacodynamic effects were evaluated using validated nociceptive and CNS test batteries.

## Methods

The study was conducted by the Centre for Human Drug Research (CHDR) in Leiden, The Netherlands, following the guidelines outlined in the Declaration of Helsinki of 1975, revised in 2013. The Medical Ethics Committee Stichting Beoordeling Ethiek Biomedisch Onderzoek (BEBO) in Assen, The Netherlands, approved this study, and it was prospectively registered in Toetsingonline: NL79589.056.21, ISRCTN30672343.

## Subjects and Study Design

Before the start of any assessments, written informed consent was obtained from all of the subjects. Healthy male and female subjects aged 18–55 underwent preliminary screening before enrollment. Essential criteria assessed for eligibility included overall good health and the absence of current or past medical conditions that could jeopardize the participants’ safety or potentially influence study outcomes.

**Figure 1:**
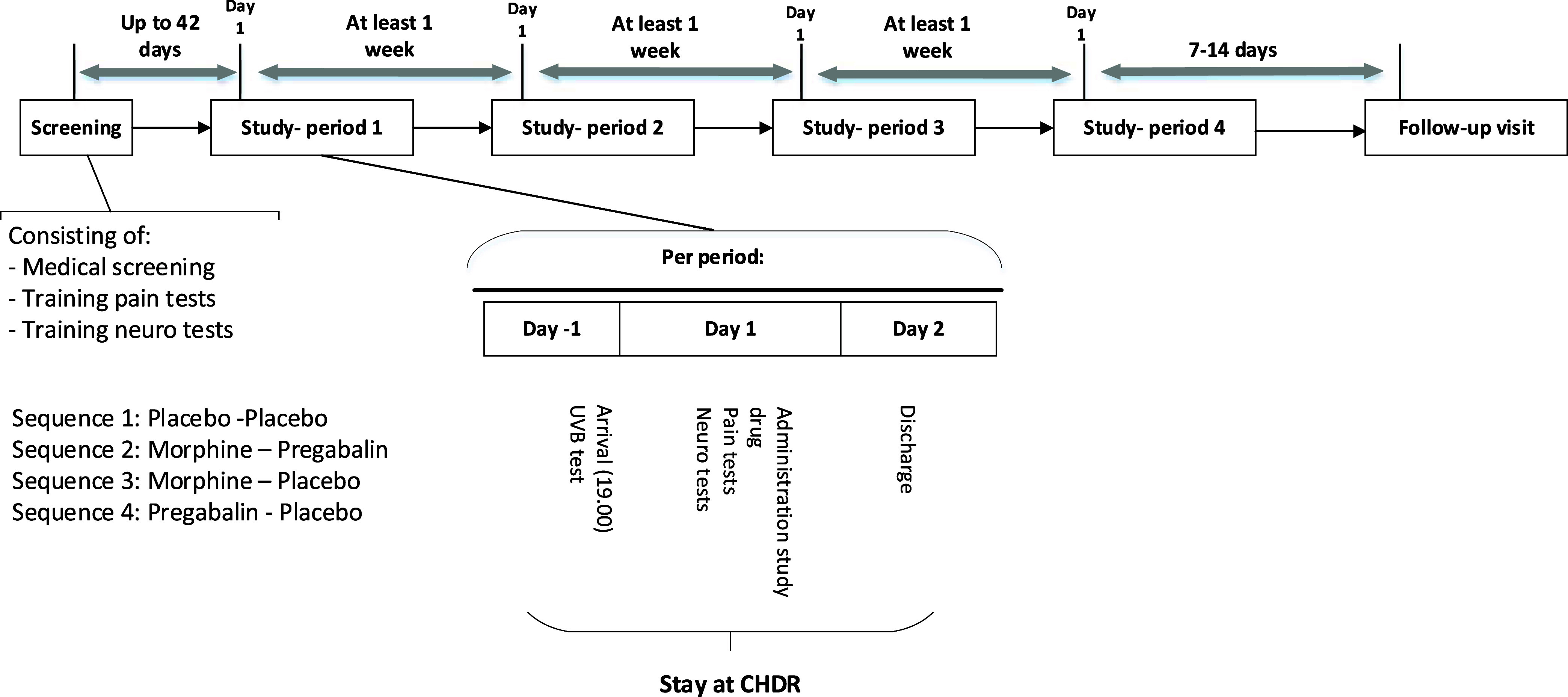
Schematic overview of the study design.

A randomized, double-blind, double-dummy, placebo-controlled, four-way crossover study was performed (Figure 1). During four separate study periods, subjects received pregabalin and morphine combined, morphine only, pregabalin only, and double placebo in a randomized order. Between each visit, there was a washout period of at least 7 days. Block randomization was produced using SAS (version 9.4, SAS Institute Inc., Cary, NC) by a statistician. Each study period included a day of admission to the clinic (day −1), a dosing and measurement day (day 1), and a day of discharge (day 2). A follow-up visit occurred 12–16 days after the fourth study period. During each study period, pharmacokinetic (PK) assessments, evoked pain tests, and CNS functioning tests were performed. Physical examinations were performed predose to reconfirm eligibility. Safety evaluations were performed throughout the study and included the evaluation of clinical chemistry and hematology blood analyses, vital signs, respiratory rate, 12-lead electrocardiograms (ECGs), and adverse event monitoring.

## Study Drugs

The combination of pregabalin and morphine was investigated to ensure consistency and translational relevance across the QSPainRelief consortium. Morphine is a widely used opioid that has been extensively characterized by other members of the consortium. Consequently, it was selected as the preferred opioid over alternatives such as oxycodone as its inclusion facilitates continued collaboration within the consortium framework. After being dosed with 300 mg of pregabalin or placebo orally (*t* = 0), subjects received either placebo or two intravenous administrations of morphine; 3 mg (at *t* = 2 h) and 7 mg (at *t* = 5 h) (Figure 1). A single dose of oral pregabalin 300 mg was chosen based on the results of four human experimental pain studies previously performed at our institute, in which this dose was found to produce significant analgesic effects with limited side effects.^22^ While single doses of pregabalin 300 mg may not be used often in clinical practice, plasma concentrations reached after the administration of pregabalin 300 mg will cover the concentrations observed at the steady state when patients are on 150 mg BID, which is very common dose taken by patients in clinical practice.^23^

Morphine was dosed intravenously as a bolus injection in 1 min and at a dose of 3 mg (*t* = 2 h) and 7 mg (*t* = 5 h). The doses were based on the morphine equivalents of “minimally therapeutic” (3 mg morphine) and “clearly therapeutic” (7 mg morphine) doses of buprenorphine, previously studied for their analgesic effects.^24^ Two doses were chosen to mimic a pharmacological range, with one representing a “minimally therapeutic” effect and the other a “clearly therapeutic” agonist effect on the mu-opioid receptor. This approach allowed for a comparison to determine whether the combination of the minimal therapeutic dose of morphine together with pregabalin has an analgesic effect equal to the clearly therapeutic dose of morphine alone. Intravenous, instead of oral, administration allowed us to better capture the concentration–analgesic effect relationship of both morphine and morphine-6-glucuronide (M6G).

Dose selection for morphine was based on established pharmacokinetic/pharmacodynamic (PKPD) models for pregabalin and morphine.^25,26^ PKPD modeling was used to forecast the analgesic effects of both drugs separately and in combination. The modeling anticipated that the proposed drug and dose combinations would amplify analgesic effects (see Supplementary Figure 1).

## Pharmacodynamic Assessments

### Evoked Pain Tests

We used a comprehensive and validated battery of evoked pain tests to determine the analgesic effects in healthy subjects. The nociceptive test battery includes the cold pressor test, electrical burst test, electrical stair test, pressure pain test, and heat pain test on normal and UVB-exposed skin.^22,27,28^ In brief, pain intensity for all tests except the heat pain test was captured using an electronic visual analogue scale (eVAS) slider: 0, “no pain” to 100, “worst pain tolerable.” For each test, two thresholds were recorded: the point at which subjects first feel pain, i.e., the pain detection threshold (PDT), and when pain becomes intolerable, i.e., the pain threshold tolerance (PTT). For the heat pain test, only PDT was recorded; PTT to heat pain was not recorded due to concerns about the potential risk of burn injuries to the subjects.

#### Cold Pressor Test

The subject’s hand was submerged in cold water to evaluate nociception and to induce conditioned pain modulation (CPM). The protocol involved placing the subject’s nondominant hand into a circulating water bath at 35 ± 0.5 °C for 2 min. After 1 min and 45 s, a blood pressure cuff on the upper arm was inflated to restrict blood flow. At the 2 min mark, the subject transferred their hand to a cold-water bath at 1.0 ± 0.5 °C. The subject then reported the PDT and PTT using the eVAS slider. The test ended when the PTT was reached or after 120 s in cold water, and the blood pressure cuff was deflated. Data was collected based on the time taken to reach PDT, PTT, or the 120 s limit.

#### Electrical Stimulation Test

The electrical stimulation method, based on Arendt-Nielsen et al.,^29^ assesses nociception from Aδ- and C-fiber sensory afferents, which transmit nociceptive signals from the periphery to the spinal cord. Two Ag-AgCl electrodes were placed on clean skin on the left tibial bone with one positioned 100 mm below the patella and the other 135 mm further down. The resistance between the electrodes was kept below 2 kΩ. For the stairs test, single pulses were delivered at a frequency of 10 Hz with a duration of 0.2 ms, controlled by a computer-operated constant current stimulator. For the burst test, each single stimulus (train of five 1 ms square wave pulses repeated at 200 Hz) was repeated five times at a frequency of 2 Hz with the same current intensity, with a random interval of 3 to 8 s between repetitions. The current intensity increased by 0.5 mA per second from 0 mA for both the stairs and burst test. Pain intensity was measured on an eVAS until PTT was reached or a maximum of 50 mA was attained.

#### Pressure Pain Test

This pressure pain induction method primarily targets nociception originating from the muscle with minimal involvement from cutaneous nociceptors. A constant pressure was applied to the gastrocnemius muscle at an increasing rate of 0.5 kPa/s using an 11 cm wide tourniquet cuff (VBM Medizintechnik GmbH, Sulz, Germany). The pressure was controlled by an electropneumatic regulator (ITV1030-31F2N3-Q, SMC Corporation, Tokyo, Japan), an analog-to-digital converter (Power1401mkII), and Spike2 software (CED, Cambridge, UK). The pressure was increased until the subject indicated their PTT or until a maximum pressure of 100 kPa was reached, after which the pressure was automatically released.

#### Heat Pain Test on Normal and UVB-Exposed Skin

During the screening visit, UVB irradiation was applied to determine each subject’s minimal erythema dose (MED). Six doses were applied to 1 cm^2^ areas on the upper back, based on the average MED for various skin phototypes, ranging from 64 to 1321 mJ/cm^2^. After 18–24 h, the MED was visually identified as the lowest UVB dose causing clear erythema. In the study, twice the subject’s UVB MED (2MED) was applied to a 3 cm^2^ area on the right scapula prior to the first pain task. PDTs were then assessed on the UVB-exposed and control areas using a thermode, which gradually increased from 32 °C at a rate of 0.5 °C/s. The subject indicated their PDT or the test stopped at 50 °C. The average of three measurements was used for analysis.

### CNS Tests

We used a comprehensive and validated test battery to evaluate neurophysiological and neurocognitive effects of the drugs in healthy subjects.^30^ This article describes the methodology of a summary of CNS tests, including the body sway test, adaptive tracking test, measurement of smooth and saccadic eye movements, the N-back test, and pharmaco-electroencephalography (-EEG) recordings.^30,31^

#### Smooth Pursuit and Saccadic Eye Movements

Smooth pursuit and saccadic eye movement analysis are commonly used to assess the (side) effects of CNS drugs.^32^ The test used was based on van Steveninck et al.^33^ Subjects were instructed to follow a horizontally moving light source on a screen positioned 58 cm away. For smooth pursuit assessment, the light moved at a steady, accelerating pace, while for saccadic eye movement evaluation, it shifted abruptly from side to side at random intervals. Each test lasted around 1 min. The smooth pursuit test recorded the percentage of time the subject’s eyes smoothly followed the target, while the saccadic test measured peak eye velocity (deg/s). Tests were conducted in a quiet, dimly lit room with only the subject present.

#### Adaptive Tracking

The adaptive tracking test evaluates (sustained) attention and executive functioning using specialized equipment and software based on TrackerUSB (Hobbs, 2004, Hertfordshire, U.K.).^34,35^ In this study, subjects used a joystick to keep a dot within a randomly moving circle on a screen. The circle’s speed increased with successful tracking and decreased with errors. Performance is measured by the percentage of time the dot remains within the circle. The test lasted 3.5 min, including a 0.5 min run-in period where no data were recorded.

#### Body Sway Test

The body sway meter is a device for assessing postural stability by recording single-plane body movements in millimeters over 2 min. At our institute, this method has been widely utilized to assess the effects of sleep deprivation,^36^ alcohol,^37^ benzodiazepines,^37,38^ and other factors. Subjects stood still with feet about 10 cm apart, hands by their sides, and eyes closed, while movement data was collected.

#### N-Back Test

The N-Back test is used to evaluate working memory. At our institute, a shortened version based on Rombouts et al.^39^ is administered in three conditions, each increasing in difficulty, with a maximum duration of 10 min. In Condition 0 (zero-back), subjects identified whether the letter presented on a computer screen is “X″ or another letter. In Condition 1 and 2 (“one-back” and “two-back”), letters were presented sequentially, followed by a black screen for 0.5 s. In Condition 1, “1-back” condition, subjects identified whether the earlier presented letter was a repetition without any other letter intervening (e.g., C, C). In Condition 2, “2-back” condition, subjects identified whether a letter was repeated with one other letter in between (e.g., C, B, C). The 3 conditions were presented in 3 blocks with increasing working memory load. Each condition has a training and a test phase.

#### Pharmaco-Electroencephalography

Pharmaco-electroencephalography (-EEG) is used to monitor any drug effects, which can be interpreted as evidence of penetration and activity in the brain. EEG recordings are performed with open and closed eyes for 5 min in each eye state (Jobert et al., 2012). EEG is continuously recorded using a 40-channel recording system (Refa-40, TMSi B.V., The Netherlands) and performed according to the guidelines of the International Pharmaco-EEG Society (IPEG). Recorded channels are band-pass filtered using a third-order Butterworth filter with cutoff frequencies at 0.5 and 45.0 Hz. The filtered signals are then divided into four second epochs. Epochs containing ocular artifacts are removed for further analysis. A power spectrum density (PSD) is calculated for each epoch and for each eye state. The resulting PSDs are then subdivided into bands, and the total power per band is calculated. The electrodes of interest for this study are Fz-Cz, Pz-O1, and Pz-O2, which is based on validation in various pharmacological studies.^40^

### Pharmacokinetic Assessments

Blood samples were drawn at: predose and at 1, 2, 4, 5, 6, 8, and 24 h postdose. Concentrations of pregabalin, morphine, and M6G were quantified using validated liquid chromatography with tandem mass spectrometry (LC-MS/MS). The lower limit of quantification was 25 ng/mL for pregabalin and 0.5 ng/mL for morphine and M6G. Reproducibility of the assays was in line with the European Medicines Agency (EMA) bioanalytical method development guideline (EMA/INS/GCP/532137/2010), with CV% < 15%.

### Statistical Analyses

A sample size of 24 participants was planned to achieve a statistical power of at least 80% and detect a 5 s difference in the cold pressor PTT between treatments with 95% confidence. Based on our experience with the cold pressor PTT and validation studies using dose levels of analgesics used in a clinical setting to treat people with pain, a 5 s difference was considered sufficient to observe an analgesic effect in the cold pressor test.^23^ In addition, our institute had data available of multiple studies with pregabalin that could be used to estimate treatment differences and test variability, i.e., key components for the sample size calculations.^22,23,41^

PD data were analyzed using SAS 9.4 in a mixed model analysis of variance, with treatment, time, period, and treatment by time as fixed factors and subject, subject by treatment and subject by time as random factors, and the average baseline measurement as a covariate. Results describe the estimate of difference (ED) and 95% confidence intervals (CI) for all the repeated measurements over the full-time course that was evaluated (i.e., predose last value prior to dosing). A *p*-value of <0.05 was considered statistically significant. This study was exploratory in nature; therefore, corrections for multiple testing were not applied.

We hypothesized that the combination of pregabalin and morphine would induce more analgesic effects with limited additional side effects compared to morphine or pregabalin. Therefore, contrasts of interest were (1) subjects receiving pregabalin and morphine combined versus placebo, (2) subjects receiving pregabalin and morphine combined versus morphine only, and (3) subjects receiving morphine only (4) or pregabalin only versus placebo. PK data were analyzed using a noncompartmental analysis in R v4.0.3. Following was reported for all treatment options: peak concentration (*C*_max_), time to peak concentration (*T*_max_), lag time, terminal half-life (*T*_1/2_) area under the curve (AUC), volume of distribution (*V*_d_), and clearance (CL). AUCs were calculated using the linear-up log-down trapezoidal method.

### Pharmacokinetic and Pharmacodynamic Model Development

The model of van Esdonk et al. on the effect of pregabalin on pain tolerance to the cold pressor test was used as a starting point for model development.^26^ Their data were best described by a one-compartment-PK model with depot and lag time connected to a turnover PD compartment. Results would determine whether we could use PK data from the combined treatment for the development of the individual PK models. Between-occasion-variability (BOV) was examined for both the PK and PD models. Individual PK posthoc estimates were used for the PD models. Model selection was based on objective function value (OFV), diagnostic plots (these include individual predictions, conditional weighted residuals (CWRES) and parameter-covariate relations), model stability, condition number, interindividual variability, and relative standard error (RSE), among others.

For modeling, NONMEM version 7.5.1 was used.^42^ For generation of plots, Perl-speaks-NONMEM (PsN) 5.3.1^43^ and R version 4.3.1 were used.^26^

## Results

### Subjects

In total, 27 subjects (44.4% male; aged 39.0 ± 16.4 years) were included and received at least one of the four treatment options (Figure 1). Twenty-four subjects completed all PK and PD assessments and received all four different treatment options.

### Pharmacodynamic Outcomes

#### Evoked Pain Tests

**Figure 2:**
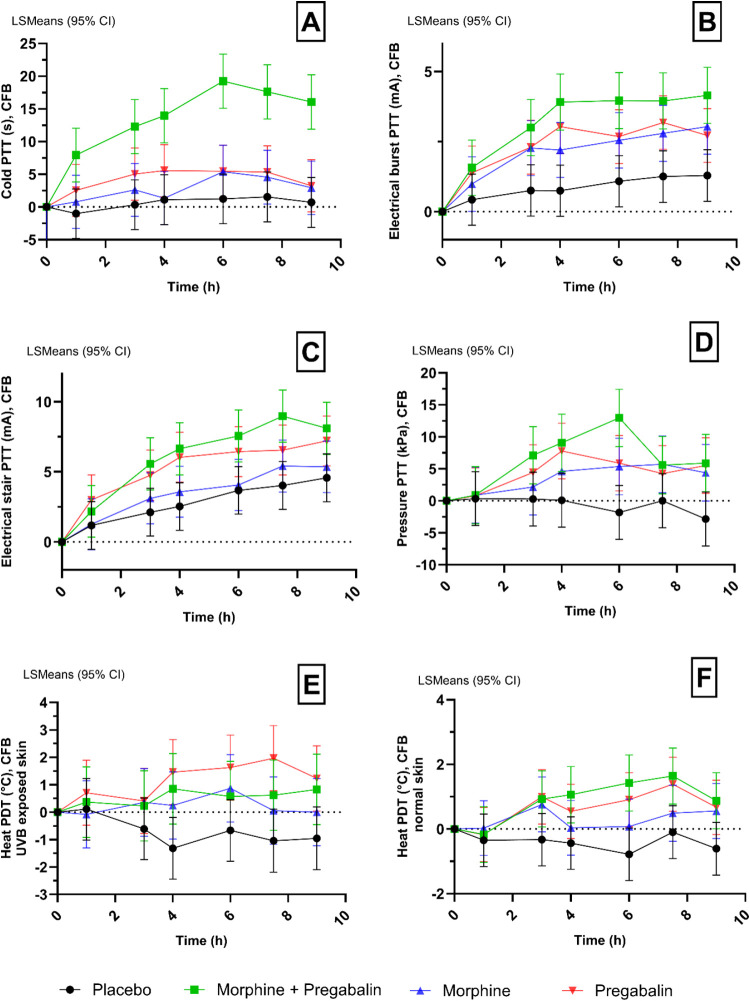
Selection of evoked pain test results in all treatment groups. Graphical presentation of a selection of the evoked pain test results in all treatment groups over time. (A) Cold pressor PTT. (B) Electrical burst PTT. (C) Electrical stair PTT. (D) Pressure pain PTT. (E) Heat pain on UVB-exposed skin PDT. (F) Heat pain on normal skin PDT. Abbreviations: PDT = pain detection threshold; PTT = pain tolerance threshold; CFB = change from baseline.

See Table 1 and Figure 2 for a summary of the pain tolerance results of the combination therapy (“pregabalin and morphine combined”), morphine only, pregabalin only, and double placebo. Refer to Supplementary Table 1 for the results of pain detection thresholds.

**Table 1:** Results of Evoked Pain Tests and CNS Functioning Tests in All Treatment Groups^a^

	outcomes (ED (95% CI); *p*-value) per contrast				
	morphine and pregabalin vs placebo	morphine vs morphine and pregabalin	pregabalin vs morphine and pregabalin	morphine vs placebo	pregabalin vs placebo
	*n* = 24	*n* = 24	*n* = 24	*n* = 24	*n* = 24
**Evoked Pain Tests**					
cold pressor PTT (sec)	***13.97 (9.27, 18.67)***	***-11.23 (−16.05, −6.42)***	***-9.81 (−14.58, −5.03)***	2.74 (−1.91, 7.38)	4.17 (−0.42, 8.75)
	*p* < 0.0001	*p* < 0.0001	*p* = 0.0001	*p* = 0.2435	*p* = 0.0745
electrical burst PTT (mA)	***2.62 (1.59, 3.65)***	***-1.28 (−2.34, −0.22)***	*-*0.92 (−1.97,0.12)	***1.34 (0.33, 2.36)***	***1.70 (0.7, 2.7)***
	*p* < 0.0001	*p* < 0.0001	*p* = 0.0824	*p* = 0.0105	*p* = 0.0012
electrical stair PTT (mA)	***3.63 (1.69, 5.58)***	***-2.71 (−4.71, −0.71)***	–0.85(−2.82, 1.13)	0.93 (−0.99, 2.85)	***2.79 (0.90, 4.68)***
	*p* = 0.0004	*p* = 0.0088	*p* = 0.3936	*p* = 0.3361	*p* = 0.0046
pressure pain PTT (kPa)	***7.32 (4.13, 10.50)***	–3.20 (−6.41, 0.01)	*-2.12 (−5.32, 1.05)*	***4.12 (1.01, 7.22)***	***5.18 (2.12, 8.25)***
	*p* < 0.0001	*p* = 0.0507	*p* = 0.1823	*p* = 0.0108	*p* = 0.0015
heat pain PDT (UVB skin) (°C)	***1.306 (0.252, 2.359)***	***-***0.303 (−1.392, 0.786)	*0.676 (−0.370, 1.723)*	***1.003 (0.002, 2.003)***	***1.982 (0.995, 2.969)***
	*p* = 0.0166	*p* = 0.5756	*p* = 0.1975	*p* = 0.0495	*p* = 0.0003
heat pain PDT (normal skin) (°C)	***1.598 (1.078, 2.119)***	***-0.761 (−1.290, −0.233)***	*-0.235 (−0.759, 0.288)*	0.837 (0.323,1.350)	1.363 (0.860,1.867)
	*p* < 0.0001	*p* = 0.0055	*p* = 0.3724	*p* = 0.0019	*p* < 0.0001
**CNS Functioning**					
smooth pursuit (%)	***-5.15 (−8.27, −2.03)***	***5.05 (1.85, 8.25)***	–1.47 (−4.64, 1.70)	–0.10 (−3.16, 2.95)	***-6.62 (−9.65, −3.60)***
	*p* = 0.0016	*p* = 0.0024	*p* = 0.3572	*p* = 0.9459	*p* < 0.0001
saccadic reaction time (sec)	–0.0090 *(*-0.0059, 0.0239)	0.0020 *(*-0.0131, 0.171)	–0.0142 (−0.0294, 0.0010)	0.0111 *(*-0.0023, 0.0245)	–0.0052 *(*-0.0186, 0.0082)
	*p* = 0.2309	*p* = 0.7881	*p* = 0.0662	*p* = 0.1034	*p* = 0.4431
adaptive tracking (%)	***-10.7 (−12.976, −8.46)***	***5.79 (3.499, 8.072)***	1.835 (−0.460, 4.130)	***-4.94 (−7.090, −2.78)***	***-8.88 (−11.036, −6.738)***
	*p* < 0.0001	*p* < 0.0001	*p* = 0.1152	*p* < 0.0001	*p* < 0.0001
body sway (mm)	***115.4%***^b^***(80.3%, 157.3%)***	***-47.6% (−56.3%, −37.2%)***	–14.7 (−28.9, 2.2)	12.8% *(*-5.2%, 34.3%)	***83.7% (54.6%, 118.2%)***
	*p* < 0.0001	*p* < 0.0001	*p* = 0.0832	*p* = 0.1726	*p* < 0.0001
N-back zero-back (nr correct-nr incorrect/total)	**-0.151 (−0.211, −0.090)**	**0.146 (0.086, 0.207)**	0.027 (−0.034, 0.087)	–0.004 (−0.063, 0.055)	**-0.124 (−0.183, −0.065)**
	*p* < 0.0001	*p* < 0.0001	*p* = 0.3846	*p* = 0.8903	*p* < 0.0001
N-back zero-back (reaction time, msec)	**36.546 (13.960, 59.131)**	–21.664 (−44.558, 1.230)	–4.657 (−27.594, 18.279)	14.882 (−7.233, 36.998)	**31.888 (10.147, 53.630)**
	*p* = 0.0019	*p* = 0.0632	*p* = 0.6862	*p* = 0.1834	*p* = 0.0047
N-back one-back (nr correct-nr incorrect/total)	**-0.168 (−0.241, −0.096)**	**0.158 (0.083, 0.232)**	0.002 (−0.072, 0.075)	–0.011 (−0.082, 0.060)	**-0.167 (−0.237, −0.096)**
	*p* < 0.0001	*p* < 0.0001	*p* = 0.9666	*p* = 0.1726	*p* < 0.0001
N-back one-back (reaction time, msec)	**51.487 (25.744, 77.229)**	**-36.388 (−62.806, −9.970)**	–7.381 (−33.622, 18.859)	15.099 (−10.099, 40.296)	**44.106 (19.099, 69.112)**
	*p* = 0.0002	*p* = 0.0077	*p* = 0.5760	*p* = 0.2355	*p* = 0.0008
N-back two-back (nr correct-nr incorrect/total)	***-0.162 (−0.240, −0.084)***	***0.127 (0.047, 0.206)***	0.036 (−0.044, 0.115)	–0.035 (−0.112, 0.041)	***-0.126 (−0.202, −0.050)***
	*p* < 0.0001	*p* = 0.0077	*p* = 0.3713	*p* = 0.3640	*p* = 0.0015
N-back two-back (reaction time, msec)	***34.658 (3.385, 65.931)***	–14.483 (−46.223, 17.258)	–28.674 (−60.327, 2.979)	20.175 (−10.417, 50.768)	5.984 (−24.327, 36.294)
	*p* = 0.0305	*p* = 0.3646	*p* = 0.0749	*p* = 0.1919	*p* = 0.6941

a Data in bold and italic denote significant effects (*P* < 0.05). Abbreviations: ED = estimate of difference; 95% CI = 95% confidence interval; PDT/PTT = pain detection/tolerance threshold; CNS = central nervous system.

b % represents the change from baseline body sway score.

##### Cold Pressor Pain Test

Pregabalin and morphine combined significantly increased tolerance to cold pressor pain compared to placebo (ED: 13.97s, 95% CI (9.27; 18.67), *p < 0.0001*) and compared to morphine (ED: −11.23s, 95% CI (−16.05; −6.42), *p < 0.0001*). No statistically significant effect was observed for either monotherapy (morphine or pregabalin) compared to placebo.

##### Electrical Burst Pain Test

Pregabalin and morphine combined significantly increased tolerance to electrical burst pain compared to placebo (ED: 2.62 mA, 95% CI (1.59; 3.65), *p < 0.0001*) and compared to morphine only (ED: −1.28 mA, 95% CI (−2.34; −0.22), *p = 0.0189*). Morphine significantly increased electrical burst pain tolerance compared to placebo (ED: 1.34 mA, 95% CI (0.33; 2.36), *p = 0.0105*), as did pregabalin compared to placebo (ED: 1.70 mA, 95% CI (0.70; 2.70), *p = 0.0012*).

##### Electrical Stair Pain Test

Pregabalin and morphine combined significantly increased tolerance to electrical stair pain compared to placebo (ED: 3.63 mA, 95% CI (1.69; 5.58), *p = 0.0004*) and compared to morphine only (ED: −2.71 mA, 95% CI (−4.70; −0.71), *p = 0.0088*). Morphine did not significantly affect electrical stair pain tolerance. However, pregabalin only did significantly increased stair pain tolerance compared to placebo (ED: 2.79 mA, 95% CI (0.90; 4.68), *p = 0.0046*).

##### Pressure Pain Test

Pregabalin and morphine combined significantly increased the pressure PTT (ED: 7.32 kPa, 95% CI (4.13; 10.50), *p < 0.0001*), but induced no significant effect compared to morphine only. Morphine and pregabalin monotherapy both also significantly increased the pressure of PTT, compared to placebo (morphine vs placebo: ED: 4.12 kPa, 95% CI (1.01; 7.22), *p = 0.0108*, and pregabalin vs placebo: ED: 5.18 kPa, 95% CI (2.12; 8.25), *p = 0.0015*).

##### Heat Pain on UVB-Exposed Skin Test

Pregabalin and morphine combined significantly increased the heat PDT on UVB-exposed skin, compared to placebo (ED: 1.306 °C, 95% CI (0.252; 2.359), *p = 0.0166*) but induced no significant effect compared to morphine only. Morphine and pregabalin as monotherapy both also significantly increased the heat PDT compared to placebo (morphine vs placebo: ED: 1.003 °C, 95% CI (0.002; 2.003), *p = 0.0495*, and pregabalin vs placebo: ED: 1.982 °C, 95% CI (0.995; 2.969), *p = 0.0003*).

##### Heat Pain on Normal Skin Test

Pregabalin and morphine combined significantly increased the heat PDT on normal skin, compared to placebo (ED: 1.598 °C, 95% CI (1.078; 2.119), *p < 0.0001*) and compared to morphine only (ED: −0.761 °C, 95% CI (−1.290; 0.233), *p = 0.0055*). Morphine and pregabalin as monotherapy both also significantly increased the heat PDT compared to placebo (morphine vs placebo: ED: 0.837 °C, 95% CI (0.323; 1.350), *p = 0.0019*, and pregabalin vs placebo: ED: 1.363 °C, 95% CI (0.860; 1.867), *p < 0.0001*).

### CNS Functioning

**Figure 3:**
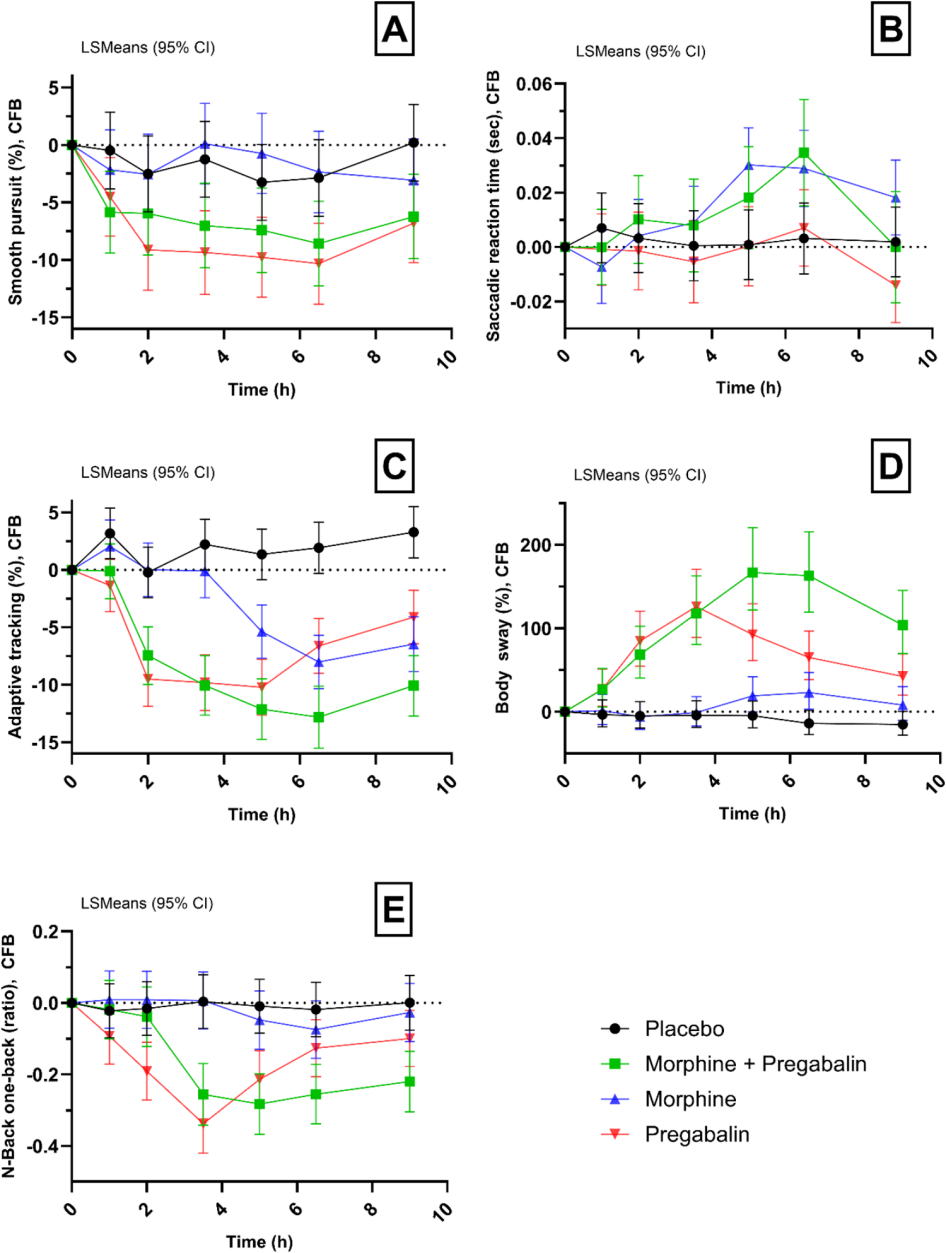
Selection of CNS functioning tests results in all treatment groups. Graphical presentation of a selection of CNS functioning test results in all treatment groups over time. (A) Smooth pursuit (%); (B) Saccadic reaction time (sec); (C) sustained attention assessed by adaptive tracking test; (D) body stability assessed by the body sway meter; and (E) working memory assessed by N-Back one-back test. Abbreviations: CNS = central nervous system; CFB = change from baseline.

See Table 2 and Figure 3A–E for a summary of the CNS tests indicative for body stability, sustained attention (adaptive tracking test), and working memory (N-back test). Outcomes of other CNS tests, including EEG recordings, are listed in Supplementary Table 2.

**Table 2:** Reported Treatment Emergent Adverse Events in All Treatment Groups

	morphine and pregabalin		morphine		pregabalin		placebo	
	*n* = 24		*n* = 24		*n* = 25		*n* = 27	
	events (*n*)	subjects (*n* (%))	events (*n*)	subjects (*n* (%))	events (*n*)	subjects (*n* (%))	events (*n*)	subjects (*n* (%))
Type of Event								
any events	189	23 (95.8)	138	24 (100.0)	89	25 (100.0)	23	13 (48.1)
ear and labyrinth disorders	1	1 (4.2)	1	1 (4.2)	-	-	-	-
eye disorders	7	5 (20.8)	-	-	3	3 (12.0)	2	2 (7.4)
gastrointestinal disorders	68	16 (66.7)	51	20 (83.3)	22	10 (40.0)	2	2 (7.4)
general disorders and administration site conditions	18	11 (45.8)	21	12 (50.0)	15	12 (48.0)	3	3 (11.1)
infections and infestations	-	-	1	1 (4.2)	-	-	4	3 (11.1)
investigations	1	1 (4.2)	1	1 (4.2)	1	1 (4.0)	-	-
metabolism and nutrition disorders	-	-	1	1 (4.2)	-	-	-	-
musculoskeletal and connective tissue disorders	10	5 (20.8)	10	7 (29.2)	1	1 (4.0)	3	3 (11.1)
nervous system disorders	57	20 (83.3)	31	16 (66.7)	37	20 (80.0)	5	5 (18.5)
psychiatric disorders	16	10 (41.7)	11	10 (41.7)	5	4 (16.0)	1	1 (3.7)
renal and urinary disorders	1	1 (4.2)	-	-	2	2 (8.0)	-	-
reproductive system and breast disorders	-	-	1	1 (4.2)	-	-	-	-
respiratory, thoracic and mediastinal disorders	8	8 (33.3)	7	4 (16.7)	1	1 (4.0)	3	1 (3.7)
skin and subcutaneous tissue disorders	1	1 (4.2)	1	1 (4.2)	1	1 (4.0)	-	-
vascular disorders	1	1 (4.2)	1	1 (4.2)	-	-	-	-

#### Smooth Pursuit and Saccadic Eye Movements

Pregabalin and morphine combined significantly decreased smooth pursuit compared to placebo (ED: −5.5%, 95% CI (−8.27; −2.03), *p = 0.0016*) and compared to morphine alone (ED: 5.05%, 95% CI (1.85; 8.25), *p < 0.0001*). Morphine alone did not affect eye movements significantly compared to the placebo. Pregabalin alone significantly decreased smooth pursuit compared to placebo (respectively ED: −6.62%, 95% CI (−9.65; −3.60), *p < 0.0001*). No significant effects of any of the drug combinations on the saccadic reaction time were observed.

#### Adaptive Tracking

Pregabalin and morphine combined significantly decreased sustained attention in the adaptive tracking test, compared to placebo (ED: −10.72%, 95% CI (−12.97; −8.46), *p < 0.0001*) and compared to morphine alone (ED: 5.786%, 95% CI (3.499; 8.072), *p < 0.0001*). Morphine alone and pregabalin alone significantly decreased adaptive tracking performance compared to placebo (respectively, ED: −4.936%, 95% CI (−7.090; −2.782), *p < 0.0001*, and ED: −8.887%, 95% CI (−11.036; −6.738), *p < 0.0001*).

#### Body Sway

Pregabalin and morphine combined significantly increased postural instability compared to placebo (ED of 115.4%, 95% CI (80.3; 157.3), *p < 0.0001*) and compared to morphine only (ED of −47.6%, 95% CI (−56.3; −37.2), *p < 0.0001*). Pregabalin alone significantly increased postural instability compared to placebo (ED of 83.7%, 95% CI (54.6; 118.2), *p < 0.0001*). No significant difference was observed between morphine alone and the placebo.

#### N-Back Test

Pregabalin and morphine combined significantly decreased the ratio (#correct – #incorrect/total) for the zero-back test, compared to placebo (ED: −0.168, 95% CI (−0.241; −0.096), *p < 0.0001*) and compared to morphine alone (ED: 0.158, 95% CI (0.083; 0.232), *p < 0.0001*). Morphine alone did not have a significant effect. Pregabalin alone compared to placebo significantly decreased the ratio compared to placebo (ED: −0.167, 95% CI (−0.237; −0.096), *p < 0.0001*). Similar effects were observed for the one- and two-back paradigms (see Table 2).

#### Pharmacokinetics

PK for pregabalin and morphine were in line with what is reported in previous studies.^22,25,26^ Mean concentrations ranged between 559.4 and 8783.2 ng/mL for pregabalin, 0.126 and 234.96 ng/mL for morphine, and 0.218 and 26.091 ng/mL for M6G. No significant differences in plasma concentration were measured for pregabalin, morphine, or morphine-6-glucuronide (M6G).

### Safety

For an overview of treatment emergent adverse events (TEAEs), see Table 2. Overall, most AEs were mild and transient and resolved without further sequelae. Most AEs were in line with the mechanism of action of the study drugs.

### Pharmacokinetic and Pharmacodynamic Model Results

Data were available from 27 included subjects. A total number of 604 PK samples were used for modeling (276 morphine and 328 pregabalin), and a total of 776 PD samples were used (214 placebo, 188 morphine, 199 pregabalin, and 175 for the combined treatment).

Morphine PK was best described as a two-compartment model. The likelihood was adjusted for limit of quantification since 12.3% of the samples were below the limit of quantification (BLQ), all at the 24 h time point.^44^ Pregabalin PK was described by a two-compartment model with a depot compartment, lag time, and allometric scaling on volumes and clearances. Adding BOV to the absorption and central clearance resulted in a significant improvement in delta-OFV of −117. No PK interactions were apparent in diagnostic plots, and thus, drug PK data from the combined treatment was used for PK model development.

The impact of morphine and pregabalin on ColdPTT was described by a turnover model. BOV on the baseline ColdPTT improved the OFV by over 100 points for both models. A declining slope of the baseline (acquired from the placebo occasion) further improved the description of the data.

The PD models were folded into a single turnover model. The turnover (K out) was estimated for both morphine and pregabalin as 0.20/h (relative standard error (RSE) of 20%). Baseline ColdPTT was estimated at 14.00 s (RSE of 19%). Best fit was achieved by the modulation of the pregabalin effect when morphine and pregabalin were present within the system (a drop in OFV of 25 points). The estimated modulation indicated a 1.8-fold increase in the pregabalin effect (RSE of 14%). This translates into an approximate 40% improvement over the additive (1 + 1) case for the ColdPTT test in healthy subjects. No biological conclusions on the direction of synergism can be drawn based on these results as the modulation of morphine instead of pregabalin provided similar data description (statistically nonsignificant delta-OFV + 3.0). Based on the PKPD model, it is concluded that the combination of morphine and pregabalin resulted in a synergistic analgesic effect on the ColdPTT test. Supplementary Figure 2 shows the Visual Predictive Checks of the PD models.

## Discussion

In this study, we performed a randomized, double-blind, placebo-controlled crossover study to evaluate the analgesic effects of pregabalin and morphine in healthy volunteers. The results show that pregabalin has an additive analgesic effect when used in combination with morphine. The increased analgesic effects observed in results for combination therapy versus either monotherapy showed that morphine and pregabalin have complementary mechanisms of action. Mechanistically, pregabalin decreases the level of release of excitatory neurotransmitters by interacting with the α-2-delta (α2δ) subunits of voltage-activated calcium channels. This inhibits the influx of cellular calcium and consequently attenuates neurotransmission, which results in therapeutic efficacy. This unique antinociceptive mechanism, distinct from morphine’s action as a mu-opioid agonist, supports the potential for increased analgesia when combining both drugs.^45^ In addition to having determined the synergistic analgesic effects of pregabalin and morphine, we used our validated CNS test battery to demonstrate that morphine and pregabalin as combination therapy did not have a worsened adverse effect profile compared to morphine or pregabalin monotherapy.

The combination of pregabalin and morphine produced several side effects, such as nausea, vomiting, headache, and somnolence. Based on these results, the combination of pregabalin and morphine might not be appropriate for opioid-naïve individuals in clinical practice. We also believe that AEs such as nausea and vomiting may have been increased because of the decision to use opioid- and pregabalin-naïve subjects. The drug combination also induced CNS effects, including sedation, as evidenced by significant changes in smooth pursuit, saccadic eye movements, and body sway. These assessments are well-established for their sensitivity to sedation caused by agents such as benzodiazepines,^46,47^ making them valuable components of the CNS test battery. Sedative adverse effects observed through the smooth pursuit assessment seemed primarily attributed to pregabalin and not worsened by the coadministration of morphine (Figure 3A). In contrast, the increased saccadic reaction time seemed to be exclusively caused by morphine (Figure 3B). Additionally, significant differences in performance on sustained attention and body stability tests were noted between the combination therapy and morphine monotherapy, with pregabalin initiating these effects and morphine prolonging them (Figure 3C,D). Based on data from other studies using this CNS test battery, we can assume that the side effects were comparable to other phase I studies assessing CNS pharmacodynamics of GABA_A_ agonists.^46−47^

This study contributes to the development of safer and more effective pain management protocols. The findings have the potential to reduce the burden of opioid-related adverse effects and addiction, offering a promising approach in the ongoing fight against the opioid epidemic while ensuring that patients with acute or chronic pain receive adequate and sustainable relief. Gabapentinoid–opioid combinations have been evaluated in various clinical studies involving patients with neuropathic pain.^49^ However, outcomes have been inconsistent, and concerns persist regarding the safety, side effects, and tolerability of low-dose pregabalin and opioids.^50,51^ The addition of gabapentin to opioid use, including oral tramadol, transdermal fentanyl, or sustained-release morphine capsules, has primarily been tested in (open-label) cohort studies in which patients were treated for neuropathic pain,^52^ cancer-related pain,^53,54^ or after an orthopedic procedure.^55^ These studies have consistently demonstrated the advantages of an opioid-nonopioid combination therapy compared to opioid monotherapy. In the literature, side effects were primarily evaluated as self-reported observation by patients.^49^ The current study stands out from previous investigations due to its placebo-controlled design, use of a healthy population, and noninvasive methodologies. The set of experimental pain and CNS tests are routinely employed in early phase drug investigations and have demonstrated the pharmacodynamic properties of a variety of drugs, including pregabalin and morphine.^22,56^ This study therefore not only supports the use of gabapentinoids as opioid–sparing-treatment but also supports the use of evoked pain tests to evaluate potential opioid–sparing treatments in early phase drug development.

Previously, *in silico*, neuronal cell models^57^ and in nonclinical studies with rats and mice also showed that the combination of morphine and pregabalin was superior in analgesia compared to either monotherapy, with limited additional side effects (personal communications). To ensure consistency and translational relevance across the different (pre)clinical phases of the consortium, the current study investigated morphine and its metabolite M6G, despite its rapid metabolism and the challenges that this might pose. For this reason, we specifically selected IV administration to bypass CYP enzyme-related first-pass metabolism, which can introduce variability in bioavailability. By controlling morphine’s bioavailability, we ensured an accurate assessment of the concentration–analgesic effect relationship for both morphine and M6G. This not only allowed us to minimize variability but also enabled us to draw meaningful comparisons to the preclinical studies conducted within the consortium. The chosen doses of morphine (7 mg IV) and pregabalin (300 mg) seem representative of effective doses commonly used in clinical practice.^8,23^ The choice for two doses of IV morphine in the study was deliberate as it allowed us to investigate the differential effects of morphine dose levels when combined with pregabalin. This approach provided critical insights into the relationship between dose escalation and analgesic efficacy, i.e., that higher doses of morphine did not enhance the therapeutic outcome (Figure 2B). The approach used in this study is not a direct translation to the patient population but serves to support research into opioid–nonopioid drug combinations. It paves the way for testing other potential drug combinations, offering valuable insights into innovative multimodal analgesic strategies.

Limitations of this study include the recruitment of healthy, therapy-naïve volunteers, and the use of single doses instead of titration regimens. These choices may have increased the incidence of AEs. Moreover, a drift (i.e., gradual increase of electrical pain tolerance thresholds over the day) was observed in the placebo treatment arm and likely in the other treatment arms as well. Is it unclear what exactly caused this drift, as this was not seen in the validation study,^22,58^ or consistently in previous studies.^24,56,59^ A reason may be fatigue of an intensive test day and treatment burden, as discussed regarding AE incidence. However, it (partly) may also be a treatment effect (Figure 2B,C).

## Conclusions

The results of this study indicate that pregabalin has an additive analgesic effect when used alongside morphine. These results corroborate previous *in silico, in vivo*, and in-patient data. Given the challenge posed by the ongoing opioid pandemic in modern healthcare, the framework of nociceptive and CNS test batteries employed in this study may serve as a valuable tool for evaluating new opioid–nonopioid drug combinations, in hopes to contribute to establishing opioid–sparing therapies.

## Supporting Information Available

Supplementary text about effects on pain detection thresholds in all treatment groups (Supplementary Table 1), effects on CNS functioning test in all treatment groups (Supplementary Table 2), effects on resting state EEG in all treatment groups (Supplementary Table 3), preclinical predicted effect on cold pressor PTT based on the integrated PKPD model of pregabalin and morphine (Supplementary Figure 1), and visual predictive check for the cold pressor PTT and PK models (Supplementary Figure 2) (PDF)

## Notes

Toetsing Online number: NL79589.056.21.
