# Supplementary material for: Application of a Nociceptive Test Battery to Assess Potential Synergy between Two Analgesics in Healthy Subjects
Source: ACS Pharmacol Transl Sci. 2025 Feb 14;8(3):819–30. doi: 10.1021/acsptsci.4c00696 (PMC11915181; doi:10.1021/acsptsci.4c00696)
Supplement: Supplementary file 1 — pt4c00696_si_001.pdf [file pt4c00696_si_001.pdf]

Supporting information

Application of a nociceptive test battery to assess potential synergy between two analgesics in healthy subjects

Wouter Alexander Bakker[1,2], Monir Bertayli[1,3], Daniël Benjamin Dumas[1,2], Jeroen Elassaiss-Schaap[3], Maria Joanna Juachon[1], Karen Broekhuizen[1], Hemme Jacob Hijma[1,2], and Geert Jan Groeneveld[1,2]*

Affiliations:

[1]Centre for Human Drug Research, Leiden, the Netherlands

[2]Leiden University Medical Centre, Leiden, the Netherlands

[3]PD-Value, Utrecht, the Netherlands

***Corresponding author:** Geert Jan Groeneveld; Centre for Human Drug Research, Zernikedreef 8, 2333CL Leiden, the Netherlands. Phone: +31 (0) 71 524 6400. E-mail: ggroeneveld@chdr.nl.

Supplementary Table 1: Effects on pain detection thresholds in all treatment groups

	**Outcomes (ED (95% CI); p-value) per contrast**				
	**Morphine and pregabalin vs Placebo**	**Morphine vs Morphine and pregabalin**	**Pregabalin vs Morphine and pregabalin**	**Morphine vs Placebo**	**Pregabalin vs Placebo**
	**n = 24**	**n = 24**	**n = 24**	**n = 24**	**n = 24**
Cold pressor PDT (sec)	***4.85 (1.46, 8.25)***	***-4.49 (7.96, -1.01)***	**-3.53 (-6.98, -0.09)**	0.37 (-2.98, 3.72)	1.32 (-1.97, 4.61)
	p = 0.0058	p = 0.0121	p = 0.0444	p = 0.8276	p = 0.4263
Electrical burst PDT (mA)	***1.20 (0.46, 1.95)***	-0.52 (-1.28, 0.24)	-0.32 (-1.08, 0.43)	0.68 (-0.05, 1.41)	***0.88 (0.16, 1.60)***
	p =0.0019	p = 0.1764	p = 0.3961	p = 0.0662	p = 0.0176
Electrical stair PDT (mA)	***3.34 (1.88, 4.81)***	***-3.18 (-4.68, -1.67)***	-1.36 (-2.85, 0.12)	0.16 (-1.28, 1.61)	***1.98 (0.56, 3.40)***
	p < 0.0001	p < 0.0001	p = 0.0716	p = 0.8208	p = 0.0070
Pressure pain PDT (kPa)	***3.34 (1.06, 5.63)***	-3.03 (-5.37, -0.68)	-2.11 (-4.43,0.22)	0.32 (-1.94, 2.58)	1.24 (-0.98, 3.46)
	p = 0.0048	p = 0.0124	p = 0.0751	p = 0,7785	p = 0.2692

Supplementary Table 2: Effects on CNS functioning test in all treatment groups

	Outcomes (ED (95% CI); p-value) per contrast				
	**Morphine and pregabalin vs Placebo**	**Morphine vs Morphine and pregabalin**	**Pregabalin vs Morphine and pregabalin**	**Morphine vs Placebo**	**Pregabalin vs Placebo**
	**n = 24**	**n = 24**	**n = 24**	**n = 24**	**n = 24**
VVLT delayed word recall number correct	***-2.0 (-3.6, -0.4)***	***2.9 (1.3, 4.5)***	***-1.1 (-2.6, 0.5)***	1.0 (-0.6, 2.5)	-0.1 (-1.6, 1.4)
	p = 0.0172	p = 0.0006	p = 0.0257	p = 0.2102	p = 0.8798
Bond & Lader VAS Alertness	***-10.6 (-13.3, -8.0)***	***6.7 (4.0, 9.4)***	***3.4 (0.7, 6.1)***	***-3.9 (-6.5, -1.3)***	***-7.2 (-9.7, -4.6)***
	p < 0.0001	p < 0.0001	p = 0.0130	p = 0.0035	p < 0.0001
Bond & Lader VAS Mood	***3.7 (1.6, 5.8)***	***-3.7 (-5.8, -1.6)***	***-2.7 (-4.8, -0.6)***	-0.0 (-2.0, 2.0)	1.0 (-1.0, 3.0)
	p = 0.0006	p = 0.0007	p = 0.0117	p = 0.9958	p = 0.3158
Bond & Lader VAS Calmness	***7.0 (4.4, 9.5)***	***-3.7 (-6.3, -1.1)***	***-3.5 (-6.1, -0.9)***	***3.3 (0.8, 5.8)***	***3.5 (0.9, 6.0)***
	p < 0.0001	p = 0.0066	p = 0.0102	p = 0.0111	p = 0.0079
Bowdle VAS (feeling high)	***0.6109 (0.4549, 0.7669)***	***-0.2899 (-0.4481, -0.1317)***	***-0.3374 (-0.4950, -0.1799)***	**0.3210 (0.1682, 0.4737)**	**0.2734 (0.1225, 0.4244)**
	p < 0.0001	p = 0.0005	p < 0.0001	p < 0.0001	p = 0.0006

Supplementary Table 3: Effects on resting state EEG in all treatment groups

	Outcomes (ED (95% CI); p-value) per contrast				
	**Morphine and pregabalin vs Placebo**	**Morphine vs Morphine and pregabalin**	**Pregabalin vs Morphine and pregabalin**	**Morphine vs Placebo**	**Pregabalin vs Placebo**
	**n = 24**	**n = 24**	**n = 24**	**n = 24**	**n = 24**
EEG Alpha-power Fz-Cz: eyes closed ((uV))	-14.8% (-30.3%, 4.1%)	4.3% (-14.7%, 27.6%)	14.30% (-7.0%, 40.4%)	-11.1% (-26.9%, 8.0%)	-2.7% (-20.2%, 18.7%)
	p=0.1155	p=0.6768	p=0.2000	p=0.2317	p=0.7869
EEG Alpha-power Fz-Cz: eyes open ((uV))	4.0% (-16.5%, 29.6%)	-4.8% (-23.6%, 18.7%)	8.30% (-13.3%, 35.2%)	-0.9% (-19.4%, 21.8%)	12.6% (-8.4%, 38.5%)
	p=0.7207	p=0.6583	p=0.4765	p=0.9267	p=0.2545
EEG Alpha-power Pz-O1: eyes closed ((uV))	**-52.8% (-62.5%, -40.5%)**	**77.3% (40.6%, 123.6%)**	19.40% (-5.8%, 51.2%)	-16.3% (-32.9%, 4.5%)	**-43.6% (-55.0%, -29.4%)**
	p=<.0001	p=<.0001	p=0.1390	p=0.1136	p=<.0001
EEG Alpha-power Pz-O1: eyes open ((uV))	-12.7% (-30.5%, 9.7%)	**34.0% (6.8%, 68.2%)**	7.70% (-14.2%, 35.3%)	17.0% (-5.6%, 45.1%)	-5.9% (-24.0%, 16.5%)
	p=0.2386	p=0.0126	p=0.5140	p=0.1474	p=0.5699
EEG Alpha-power Pz-O2: eyes closed ((uV))	**-50.6% (-59.9%, -39.0%)**	**72.2% (39.7%, 112.3%)**	23.30% (-0.5%, 52.7%)	-14.9% (-30.4%, 4.0%)	**-39.1% (-50.3%, -25.2%)**
	p=<.0001	p=<.0001	p=0.0549	p=0.1137	p=<.0001
EEG Alpha-power Pz-O2: eyes open ((uV))	-16.7% (-31.6%, 1.5%)	**32.8% (9.1%, 61.6%)**	7.10% (-12.0%, 30.3%)	10.6% (-8.6%, 33.9%)	-10.8% (-25.8%, 7.3%)
	p=0.0695	p=0.0056	p=0.4859	p=0.2927	p=0.2195
EEG Beta-power Fz-Cz: eyes closed ((uV))	-6.3% (-18.3%, 7.4%)	2.8% (-10.2%, 17.7%)	11.30% (-3.1%, 27.9%)	-3.7% (-15.6%, 9.9%)	4.3% (-8.8%, 19.3%)
	p=0.3423	p=0.6847	p=0.1271	p=0.5705	p=0.5345
EEG Beta-power Fz-Cz: eyes open ((uV))	-3.6% (-18.4%, 13.8%)	-0.1% (-15.4%, 18.0%)	9.30% (-7.5%, 29.2%)	-3.7% (-17.7%, 12.6%)	5.4% (-9.8%, 23.1%)
	p=0.6582	p=0.9920	p=0.2888	p=0.6303	p=0.5041
EEG Beta-power Pz-O1: eyes closed ((uV))	**-38.7% (-49.4%, -25.6%)**	**36.1% (12.0%, 65.4%)**	**40.90% (15.5%, 71.9%)**	-16.5% (-30.9%, 0.8%)	-13.6% (-28.7%, 4.7%)
	p=<.0001	p=0.0025	p=0.0010	p=0.0595	p=0.1325
EEG Beta-power Pz-O1: eyes open ((uV))	**-23.3% (-36.6%, -7.1%)**	**32.1% (8.6%, 60.8%)**	**34.00% (10.3%, 63.0%)**	1.4% (-15.4%, 21.5%)	2.9% (-14.2%, 23.2%)
	p=0.0075	p=0.0062	p=0.0040	p=0.8784	p=0.7557

EEG Beta-power Pz-O2: eyes closed ((uV))	**-34.3% (-45.5%, -20.7%)**	**25.3% (3.7%, 51.3%)**	**39.40% (14.9%, 69.1%)**	**-17.7% (-31.4%, -1.1%)**	-8.4% (-24.0%, 10.4%)
	p=<.0001	p=0.0202	p=0.0010	p=0.0377	p=0.3519
EEG Beta-power Pz-O2: eyes open ((uV))	**-19.0% (-32.5%, -2.8%)**	**22.3% (1.6%, 47.2%)**	**28.00% (6.4%, 54.1%)**	-0.9% (-16.6%, 17.8%)	3.7% (-12.6%, 23.1%)
	p=0.0242	p=0.0338	p=0.0098	p=0.9168	p=0.6697
EEG Delta-power Fz-Cz: eyes closed ((uV))	**234.9% (169.8%, 315.8%)**	**-65.9% (-72.5%, -57.6%)**	**-24.50% (-39.5%, -5.7%)**	14.2% (-7.4%, 40.9%)	**153.0% (104.2%, 213.4%)**
	p=<.0001	p=<.0001	p=0.0141	p=0.2093	p=<.0001
EEG Delta-power Fz-Cz: eyes open ((uV))	**205.3% (145.2%, 280.2%)**	**-63.5% (-70.7%, -54.4%)**	-9.70% (-27.7%, 12.8%)	11.5% (-9.3%, 37.1%)	**175.7% (123.5%, 240.1%)**
	p=<.0001	p=<.0001	p=0.3639	p=0.2967	p=<.0001
EEG Delta-power Pz-O1: eyes closed ((uV))	**181.9% (115.4%, 268.9%)**	**-63.5% (-72.2%, -52.1%)**	-14.60% (-35.2%, 12.7%)	2.9% (-20.8%, 33.8%)	**140.9% (84.5%, 214.5%)**
	p=<.0001	p=<.0001	p=0.2594	p=0.8263	p=<.0001
EEG Delta-power Pz-O1: eyes open ((uV))	**163.6% (104.1%, 240.5%)**	**-64.0% (-72.2%, -53.5%)**	0.50% (-22.5%, 30.4%)	-5.2% (-25.7%, 20.9%)	**164.9% (108.4%, 236.8%)**
	p=<.0001	p=<.0001	p=0.9706	p=0.6622	p=<.0001
EEG Delta-power Pz-O2: eyes closed ((uV))	**194.8% (123.5%, 288.7%)**	**-67.4% (-75.3%, -56.8%)**	-15.80% (-36.6%, 11.9%)	-3.8% (-26.5%, 26.0%)	**148.2% (88.8%, 226.3%)**
	p=<.0001	p=<.0001	p=0.2303	p=0.7761	p=<.0001
EEG Delta-power Pz-O2: eyes open ((uV))	**156.9% (94.2%, 239.9%)**	**-63.2% (-72.3%, -51.1%)**	8.90% (-18.4%, 45.3%)	-5.4% (-27.3%, 23.1%)	**179.9% (114.6%, 265.1%)**
	p=<.0001	p=<.0001	p=0.5551	p=0.6751	p=<.0001
EEG Gamma-power Fz-Cz: eyes closed ((uV))	1.0% (-11.3%, 15.0%)	0.3% (-12.0%, 14.4%)	1.20% (-11.5%, 15.7%)	1.3% (-10.7%, 15.0%)	2.2% (-10.2%, 16.2%)
	p=0.8791	p=0.9578	p=0.8641	p=0.8329	p=0.7404
EEG Gamma-power Fz-Cz: eyes open ((uV))	12.9% (-5.9%, 35.5%)	-2.3% (-18.8%, 17.5%)	-3.60% (-19.9%, 16.2%)	10.3% (-7.0%, 30.9%)	8.9% (-8.2%, 29.2%)
	p=0.1870	p=0.8014	p=0.6971	p=0.2543	p=0.3226
EEG Gamma-power Pz-O1: eyes closed ((uV))	-9.7% (-33.5%, 22.7%)	-2.3% (-28.2%, 32.8%)	**54.20% (12.5%, 111.3%)**	-11.8% (-34.5%, 18.9%)	**39.2% (2.4%, 89.3%)**
	p=0.5088	p=0.8793	p=0.0080	p=0.4038	p=0.0351
EEG Gamma-power Pz-O1: eyes open ((uV))	-7.3% (-36.9%, 36.2%)	7.5% (-27.3%, 59.1%)	43.90% (-2.7%, 112.8%)	-0.3% (-30.7%, 43.3%)	33.4% (-7.2%, 91.7%)
	p=0.6934	p=0.7102	p=0.0677	p=0.9857	p=0.1174

EEG Gamma-power Pz-O2: eyes closed ((uV))	-6.6% (-29.9%, 24.4%)	-10.6% (-33.0%, 19.3%)	48.10% (10.3%, 98.9%)	-16.5% (-36.8%, 10.4%)	**38.4% (4.1%, 83.8%)**
	p=0.6348	p=0.4404	p=0.0098	p=0.2012	p=0.0258
EEG Gamma-power Pz-O2: eyes open ((uV))	-5.2% (-29.9%, 28.4%)	-12.7% (-35.7%, 18.7%)	**41.80% (4.3%, 92.9%)**	-17.2% (-37.7%, 10.2%)	34.5% (1.0%, 79.2%)
	p=0.7272	p=0.3790	p=0.0269	p=0.1905	p=0.0429
EEG Theta-power Fz-Cz: eyes closed ((uV))	**31.2% (9.9%, 56.6%)**	**-27.1% (-38.9%, -13.0%)**	6.40% (-11.3%, 27.7%)	-4.3% (-19.5%, 13.8%)	**39.6% (17.0%, 66.5%)**
	p=0.0032	p=0.0007	p=0.4993	p=0.6124	p=0.0004
EEG Theta-power Fz-Cz: eyes open ((uV))	**38.3% (6.6%, 79.4%)**	**-26.3% (-43.3%, -4.3%)**	10.50% (-15.2%, 44.0%)	1.9% (-20.3%, 30.2%	**52.8% (19.1%, 96.0%)**
	p=0.0155	p=0.0228	p=0.4531	p=0.8796	p=0.0012
EEG Theta-power Pz-O1: eyes closed ((uV))	**72.4% (42.2%, 109.0%)**	**-32.4% (-44.2%, -18.1%)**	2.80% (-15.5%, 25.1%)	16.5% (-3.3%, 40.4%)	**77.3% (46.4%, 114.6%)**
	p=<.0001	p=0.0001	p=0.7762	p=0.1067	p=<.0001
EEG Theta-power Pz-O1: eyes open ((uV))	**84.3% (43.4%, 136.7%)**	-43.1% (-55.8%, -26.9%)	2.40% (-20.4%, 31.7%)	4.8% (-17.2%, 32.5%)	**88.7% (48.9%, 139.1%)**
	p=<.0001	p=<.0001	p=0.8511	p=0.6922	p=<.0001
EEG Theta-power Pz-O2: eyes closed ((uV))	**75.8% (41.5%, 118.4%)**	**-37.5% (-49.7%, -22.4%)**	2.90% (-17.5%, 28.2%)	9.8% (-10.9%, 35.4%)	**80.8% (45.8%, 124.2%)**
	p=<.0001	p=<.0001	p=0.7979	p=0.3739	p=<.0001
EEG Theta-power Pz-O2: eyes open ((uV))	**72.0% (32.0%, 124.1%)**	**-45.1% (-57.8%, -28.5%)**	3.10% (-20.9%, 34.3%)	-5.5% (-26.2%, 21.0%)	**77.3% (38.3%, 127.2%)**
	p=0.0001	p=<.0001	p=0.8193	p=0.6453	p=<.0001

Supplementary Figure 1: Preclinical predicted effect on cold pressor PTT based on the integrated PKPD model of pregabalin and morphine

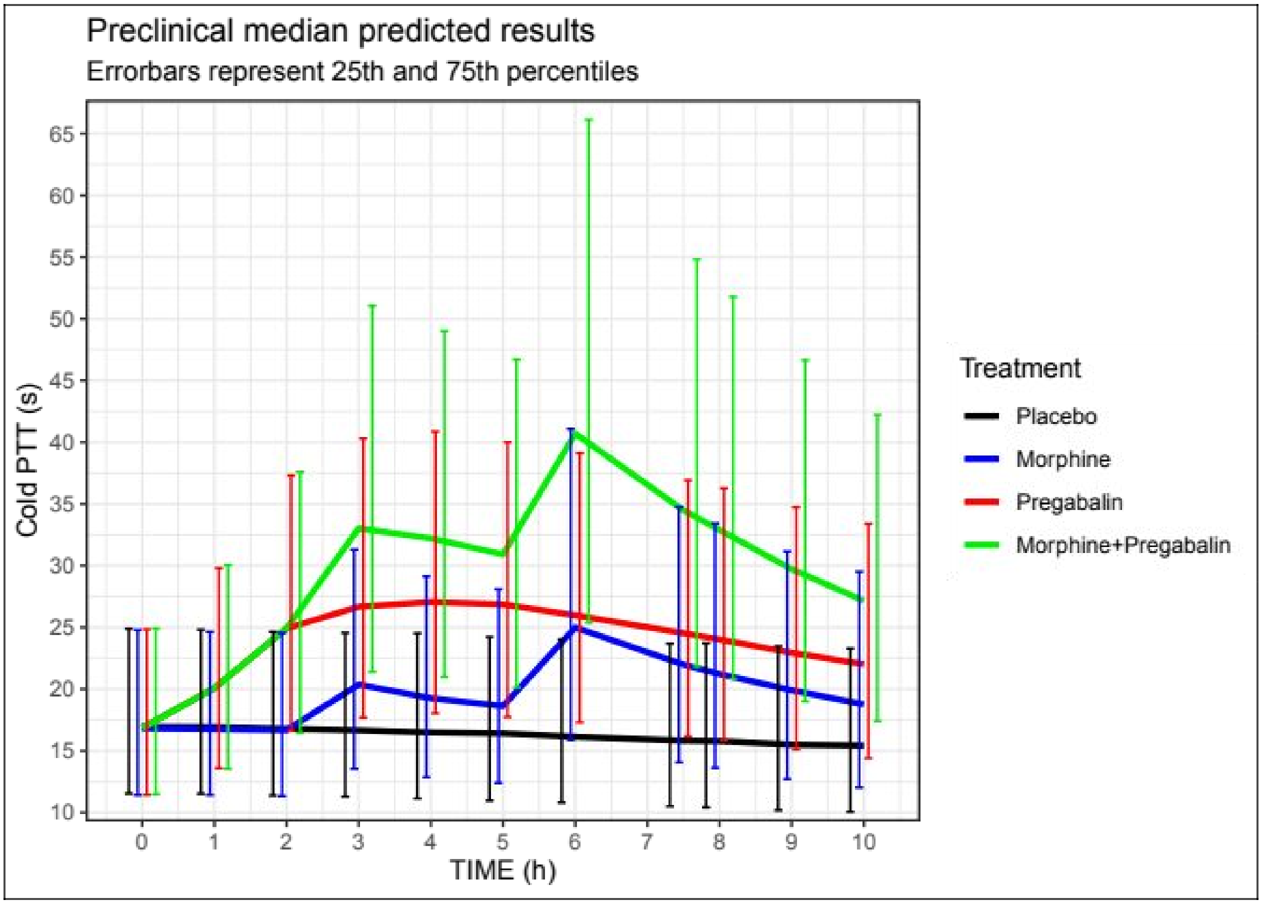

Black line is the predicted placebo response, blue line the proposed morphine dose of 3mg IV at t = 2 h and 7mg IV at t = 5 h, the red line the single oral dose of pregabalin 300mg at t=0 and green line the combination treatment of above-mentioned doses and drugs. Abbreviations: PTT = pain tolerance threshold; h = hour; s = second; mg = milligram.

Supplementary Figure 2: Visual Predictive Check for the cold pressor PTT and PK models

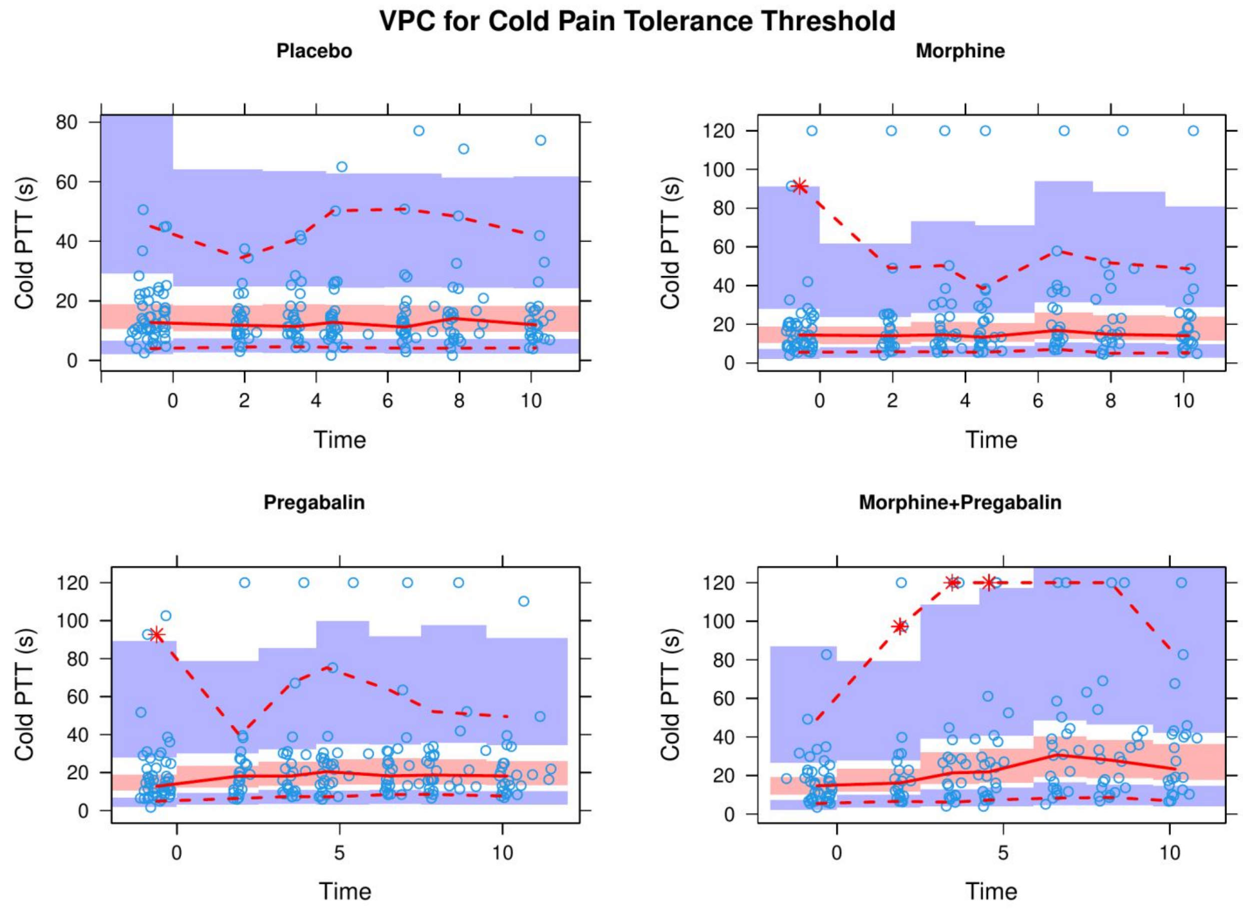

VPC = Visual Predictive Check
